# Ethnic differences in kidney function in childhood: the Born in Bradford Cohort Renal Study

**DOI:** 10.12688/wellcomeopenres.17796.2

**Published:** 2023-08-25

**Authors:** Nida Ziauddeen, Robin F. Jeffrey, Dagmar Waiblinger, Simon D.S. Fraser, Nisreen A. Alwan, Ho M. Yuen, Rafaq Azad, Dan Mason, John Wright, Richard J.M. Coward, Paul J. Roderick

**Affiliations:** 1 School of Primary Care, Population Sciences and Medical Education, Faculty of Medicine, University of Southampton, Southampton, UK; 2 NIHR Applied Research Collaboration Wessex, Southampton, UK; 3 Bradford Institute for Health Research, Bradford Royal Infirmary, Bradford, UK; 4 NIHR Southampton Biomedical Research Centre, University of Southampton and University Hospital Southampton NHS Foundation Trust, Southampton, UK; 5 Bristol Renal, Bristol Medical School, University of Bristol, Bristol, UK

**Keywords:** South Asian, kidney function, development

## Abstract

**Background:** Endstage kidney failure rates are higher in South Asians than in White Europeans. Low birth weight is associated with adult chronic kidney disease and is more common in South Asians. Foetal kidney size was smaller in South Asians in the Born in Bradford (BiB) birth cohort. As part of BiB follow up, we aimed to investigate if there were ethnic differences in kidney function and blood pressure in early childhood and whether this was different by foetal kidney size.

**Methods:** Serum creatinine, cystatin C, urea, and urinary albumin to creatinine ratio (ACR), protein to creatinine ratio (PCR) and retinol binding protein (RBP) were analysed in blood and urine samples from those who participated in the BiB follow-up at 7–11 years. Ethnicity was categorised by parental self-report as White European and South Asian. Estimated glomerular filtration rate (eGFR) was calculated using Schwartz, and cystatin C Zappitelli and Filler equations. Linear regression was used to examine the association between ethnicity and eGFR, PCR and blood pressure.

**Results:** 1591 children provided blood (n=1403) or urine (n=625) samples. Mean eGFR was 92 ml/min/1.73m ^2^ (standard deviation (SD) 9) using Schwartz (n=1156) and 94 (SD 11) using Zappitelli (n=1257). CKD prevalence was rare (1 with eGFR <60 ml/min/1.73m ^2^, 14 (2.4%) had raised ACR (>2.5 mg/mmol in boys/3.5 mg/mmol in girls). Diastolic blood pressure was higher in South Asian children (difference 2.04 mmHg, 95% CI 0.99 to 3.10) but was not significant in adjusted analysis. There was no evidence of association in adjusted models between ethnicity and any eGFR or urinary measure at this age.

**Conclusions:** There was no evidence of significant ethnic differences in kidney function at pre-pubertal age despite differences in kidney volume at birth. Longitudinal follow-up is required to track ethnic patterns in kidney function and blood pressure as children develop through puberty.

## Introduction

The incidence of end stage kidney failure (ESKF) is higher and occurs at a younger age in adults of South Asian heritage compared to White Europeans ^1, 2^. The reasons are not fully understood but Type 2 diabetes and diagnosed hypertension are more common in South Asian adults ^3, 4^ and blood pressure (BP) is higher at similar levels of obesity ^3– 5^. Whilst two primary care studies in the UK reported decreased stage 3 chronic kidney disease (CKD) prevalence in South Asians, the prevalence was higher in South Asians at more advanced stages ^3, 4^. Albuminuria is more common in South Asians ^6^. Data on differences in CKD progression to ESKF in South Asians and competing risk of mortality are limited ^7^.

The excess incidence of advanced CKD has major implications for disease burden internationally, as the South Asian diaspora increases and ages, and globally with over 1 billion people on the Indian subcontinent. CKD is an independent factor for cardiovascular disease, and understanding causation is key to prevention of CKD and its complications in such populations.

Kidney development is one area of focus as low birth weight (LBW) is more common in South Asians ^8, 9^ and is associated with adult chronic diseases such as hypertension and Type 2 diabetes that are associated with CKD ^10– 12^. A systematic review and a subsequent study found an association between LBW and CKD though there were no specific data on South Asians ^13, 14^. One hypothesis to explain such CKD risk is that intra-uterine growth retardation (IUGR), especially in the third trimester when foetal kidney development occurs, leads to reduction in nephron number ^15, 16^ and lower kidney size at birth ^17, 18^. As nephron number is fixed at birth ^15^, compensatory glomerular hypertrophy, hyperfiltration and hypertension, could lead to further reduction in nephrons, and susceptibility to kidney damage ^19, 20^. IUGR has been found to be associated with albuminuria in infancy ^21, 22^, with similar estimated Glomerular Filtration Rate (eGFR) at age 2 despite smaller kidney size, suggesting hyperfiltration ^22^, and with diastolic blood pressure (dBP) at age 6 ^23^.

The Born in Bradford (BiB) birth cohort is a prospective longitudinal multi-ethnic birth cohort study that aims to examine the impact of environmental, psychological and genetic factors on health and wellbeing in a deprived population. The full BiB cohort recruited 12453 women involving 13776 pregnancies (13858 births) between 2007 and 2010 ^24^. ln a renal subset of BiB (n= 1587), we showed that mean foetal kidney volume at 34 weeks gestation was 16% lower in South Asian babies ^25^. In the Generation R cohort in the Netherlands, lower foetal kidney volume was independently associated with reduced eGFR and kidney volume at age 6 ^26^. In the BiB cohort, dBP was higher in Pakistani children at age 4/5 years ^27, 28^.

The BiB cohort is an ideal context in which to explore the origins of ethnic differences in CKD. This study aimed to investigate whether there were ethnic differences in kidney function and damage and BP in childhood (age 7–11 years) and whether this was different by foetal kidney size.

## Methods

### Baseline recruitment in pregnancy

Women were recruited to the BiB cohort study while attending for their glucose tolerance test (OGTT), offered to all pregnant women registered at Bradford Royal Infirmary at 24–28 weeks of gestation.

BiB children aged 7–11 years and their families were followed up using a multi-method approach between 2017 and 2020 ( **BiB Growing Up Study**) ^29^. Detailed parent and child questionnaires, BP, anthropometry and blood samples were collected. Written informed consent was collected for the follow-up and for continued routine data linkage.

### Renal ultrasound sub-study recruitment in pregnancy

A renal sub-study was nested within the full BiB cohort at baseline. Women who were attending for the OGTT at 26–28 weeks of gestation, had consented for the main BiB study and completed the baseline questionnaire, were invited to undertake a further foetal ultrasound scan (USS) at 34 weeks for standard anthropometrics and foetal renal dimension measurement. Data on renal ultrasound was available for 1802 women, details of recruitment and ultrasonography were published previously ^25^.

### Follow-up population

Recruitment for the renal US sub-study follow-up commenced in January 2018 and was initially limited to children whose mothers had participated in the baseline renal sub-study and had a foetal ultrasound, termed the **BiB renal ultrasound follow-up.** This was expanded to the broader cohort due to lower than expected uptake and children who had blood sampling as part of their BiB Growing-Up follow-up were included. From January 2019, all participants who agreed to blood sampling were also asked to provide a urine sample. We called this the **BiB extended renal follow-up.** Study recruitment ended in March 2020.

**Figure 1.**
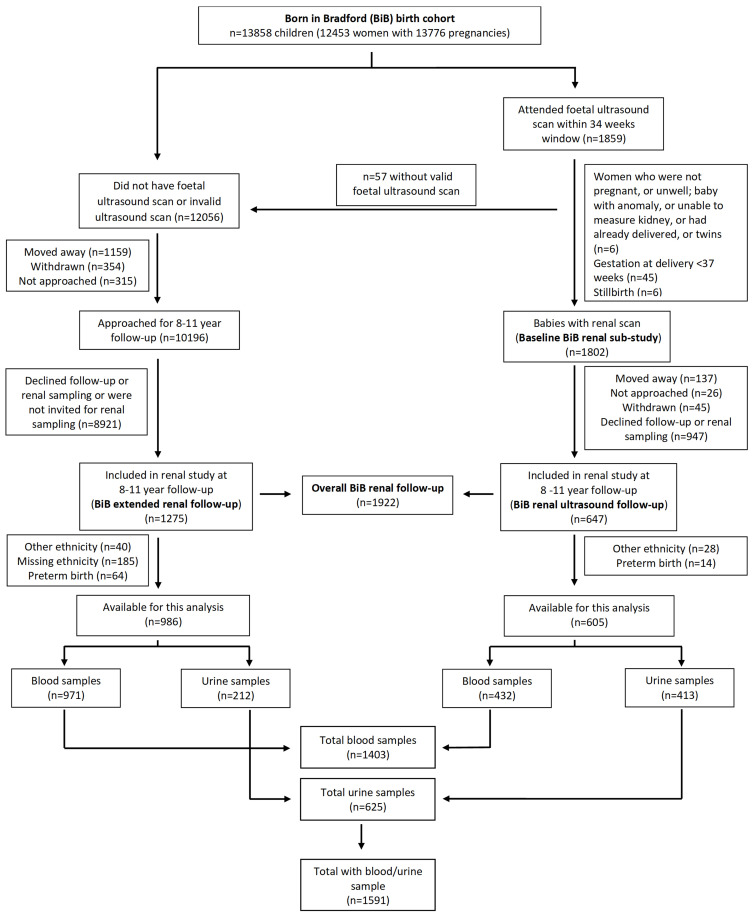
CONSORT flow chart of recruitment from the BiB Growing Up study.

For this analysis we only included those with a White European or South Asian ethnicity, who were born at term and who provided blood or urine measures. Figure 1 shows the pattern of recruitment.

Participants’ height and weight were measured without shoes and in light clothing. BP measurements were taken at the brachial artery preferably on the left arm, twice with skinfold measurements in between the first and second measurement, using an Omron electronic monitor 705-CPII. The appropriate cuff size was used. Children were seated for two minutes prior to the BP measurement. One BP measurement was recorded per child consistent with other studies undertaken within a school setting ^30, 31^. Non-fasting blood samples were taken. First morning urine samples were taken at home and transported to the Bradford Royal Infirmary laboratory. Data collection was undertaken by trained staff in schools, clinics or at the participant’s home using standard operating procedures.

### Laboratory methods

Blood samples were analysed for creatinine, urea and cystatin C. Creatinine and urea were analysed on Beckman AU650. The creatinine calibrator is traceable to the Isotope Dilution Mass Spectrometry (IDMS) reference method used by the National Institute of Standards and Technology Standard Reference Material 967. The coefficient of variation (CV) for serum creatinine was 5.7%. Cystatin C was measured using particle enhanced immunoturbidimetric assay on Roche/Hitachi cobas c systems. The assay is standardised against the ERM-DA471/IFCC certified reference material for cystatin C. CVs were 1.6–2.6% in the range assayed.

Urine samples were analysed for protein, albumin, creatinine and retinol binding protein (RBP). The urine protein method was based on Pyrogallol red, urine albumin using a turbidometric assay and urine creatinine was an enzymatic assay, all measured on Beckman AU680. The limit of detection for urine albumin was 3mg/L. CVs were 2.6% for creatinine, 8% for albumin and 7.5% for protein. RBP was measured using the immunonephelometry method on a Siemens Atellica630 Neph Nephelometer. The reference range is <15mg/L, the limit of detection is 3mg/L and CV was 5.3%.

### Renal and BP outcomes

Outcomes included eGFR, urinary albumin to creatinine ratio (ACR), urinary protein to creatinine ratio (PCR), RBP and systolic blood pressure (sBP) and dBP. Each outcome was considered separately and all (except RBP) were assessed as continuous variables. 

eGFR was calculated using four published equations as follows:

- Schwartz creatinine only = 41.3*(height (m)/ S _cr_) ^32^
- 2012 CKiD Schwartz combined serum creatinine and cystatin C = 39.8 x [(height (m ^2^)/S _cr_(mg/dl)] ^0.456 ^x [1.8/cystatin C (mg/L)] ^0.418 ^x [30/blood urea nitrogen (mg/dl)] ^0.079^ x [1.076 ^male^] x [height (m)/1.4] ^0.179^ ^32^
- Zappittelli cystatin C only = 75.94/[cystatin C^1.17] ^33^
- Filler cystatin C only log(eGFR) = 1.962 + (1.123*log(1/cystatin C)) ^34^

Child height required to calculate eGFR using both Schwartz equations was measured at the same visit as the blood sample in 52.0%, the height measurement was standardised to time of sample using centile charts if not measured at the same visit.

Urine albumin was only detectable in 296/595, 49.7% of the sample.

### Exposure

Ethnicity was self-reported by mothers at baseline interview, with participants given response options based on the UK Office of National Statistics guidance ^35^. Mothers of other ethnicities (n=68) were excluded due to small numbers. Ethnicity was categorised as White British and South Asian (predominantly of Pakistani origin).

### Covariates

*Maternal:* body mass index (BMI), age, parity, smoking, alcohol consumption, measures of socio-economic position (maternal highest educational attainment, housing tenure [buying/own house and renting or other related], and employment status), marital status (taken at first antenatal booking) and gestational diabetes (during pregnancy).

**Figure 2.**
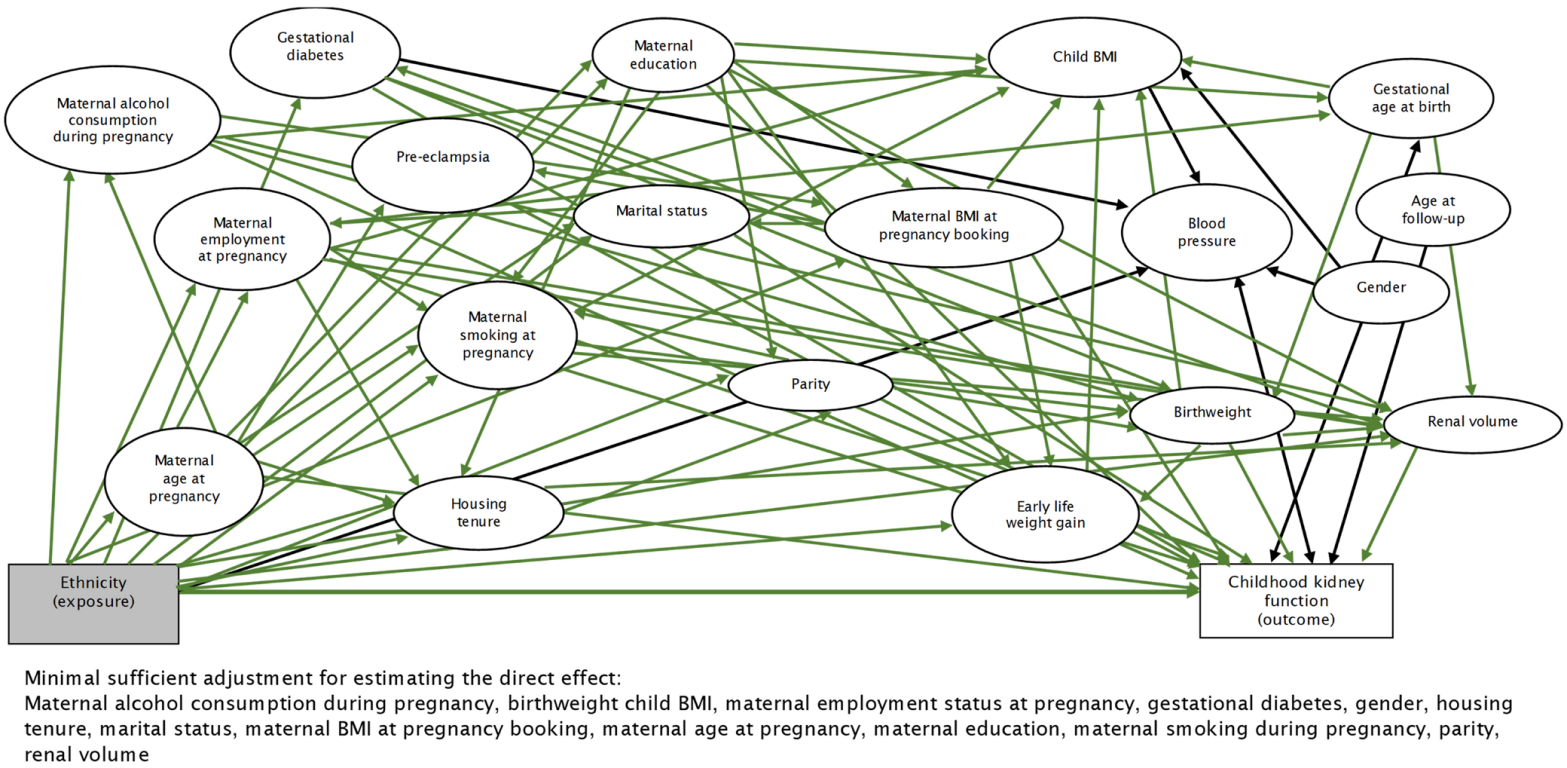
Directed acyclic graph illustrating the relationship between ethnicity and childhood kidney function. BMI=body mass index.

*Child*: Birth weight, gender and gestational age at birth; age and measured weight and height at follow-up. Measured weight and height were used to calculate BMI and body surface area (BSA). BMI was converted to age- and sex- adjusted z-scores according to the UK 1990 growth reference charts. BSA was calculated using the Du Bois formula ^36^ (BSA = weight (kg) ^0.425^ x height (cm) ^0.725^ x 0.007184). BP was not identified as a covariate to adjust for from the directed acyclic graph (DAG) but as the direction of this relationship is not known, we included BP as a covariate ( Figure 2). 

### Statistical analysis

All analysis was carried out in Stata v17 (RRID:SCR_012763) ^37^. The selection of covariates into the multivariable models were guided by a DAG constructed using DAGitty v3.0 ^38^.

Basic descriptive statistics are presented for the overall sample and stratified by ethnicity. Univariable comparisons were carried out using t-test (continuous variables) and chi square test (categorical variables). Pearson correlation was used to assess the relationships between the outcome measures (eGFR, ACR, PCR and BP). Linear regression was used to examine the association between ethnicity and the outcome measures, except for the ACR detectable binary outcome where logistic regression was used with the ACR result outcome conditional on a positive ACR detectable outcome.

Covariates were added sequentially to form multivariable models:

Model 1: maternal age, maternal education, housing tenure, employment status, marital status and child age at kidney function measurement (socio-demographic);

Model 2: additionally adjusted for maternal BMI, parity, smoking and alcohol consumption in pregnancy, and gestational diabetes (maternal/pregnancy);

Model 3: further adjusted for birthweight, BSA (kidney outcomes) or BMI (BP outcomes), gender, sBP and dBP (kidney outcomes) (fully adjusted).

We adjusted for foetal renal volume in the sub-sample that had renal ultrasound scans in pregnancy. A statistical significance level of 0.01 with 95% confidence interval (CI) was used.

Data on covariates such as marital status, employment status and housing tenure collected through baseline questionnaires were missing in women who did not complete the questionnaire (n=1057 of 12453 women from the BiB baseline sample). Some of these women completed an additional questionnaire at follow-up which reduced the missing data on ethnicity and educational attainment but not on other covariates. It was not possible to impute missing data as data were missing on all variables collected using the baseline questionnaire.

We undertook sensitivity analysis to explore the impact of differences in timing of height measurement and blood sampling for the Schwartz eGFR formulae by comparing estimates using standardised height, height as measured without accounting for timing between sampling and measurement, and sample restricted to those with height measurement and blood sample at the same. We also undertook sensitivity analysis replacing BSA with BMI in eGFR, ACR and PCR models.

### Ethical approval

Ethical approval was granted by the Bradford Research Ethics Committee (ref. 07/H1302/112 for original cohort and 16/YH/0320 (IRAS 207543) for Growing Up).

## Results

In total, 1591 children were available for this analysis, 1403 provided a blood sample and 625 a urine sample ( Figure 1). Overall, 605 from the baseline BiB renal ultrasound sub-study were eligible and provided either a blood and/or urine sample, a response rate of 34% ( Figure 1, Table 1).

**Table 1.** Selected descriptive variables from the baseline data to assess difference in sample characteristics at follow-up by response *.

	All renal follow-up	BiB renal ultrasound follow-up				BiB extended renal follow-up			
		Follow-up			Non-response to renal follow-up	Follow-up			Non- response
		Blood or urine	Blood	Urine		Blood or urine	Blood	Urine	
	n (%)	n (%)	n (%)	n (%)	n (%)	n (%)	n (%)	n (%)	n (%)
N	1591	605	432	413	1155	986	971	212	4617
Mother’s ethnic group									
White	492 (30.9)	271 (44.8)	190 (44.0)	185 (44.8)	678 (58.7)	221 (22.4)	218 (22.5)	47 (22.2)	1336 (34.6)
South Asian	1099 (69.1)	334 (55.2)	242 (56.0)	228 (55.2)	403 (34.9)	765 (77.6)	753 (77.6)	165 (77.8)	2347 (60.8)
Derived equivalized mother’s education									
None (<5 GCSE or equivalent)	392 (24.7)	121 (20.0)	81 (18.8)	78 (18.9)	173 (15.0)	271 (27.5)	267 (27.5)	45 (21.2)	831 (21.6)
School (≥5 GCSE or equivalent)	454 (28.6)	177 (29.3)	134 (31.0)	118 (28.6)	377 (32.7)	277 (28.1)	274 (28.3)	59 (27.8)	1155 (30.0)
Further and higher (A level or equivalent or higher)	638 (40.1)	265 (43.8)	188 (43.5)	190 (46.0)	522 (45.3)	373 (37.9)	367 (37.8)	94 (44.3)	1565 (40.6)
Other (Other, Overseas, Unknown)	106 (6.7)	42 (6.9)	29 (6.7)	27 (6.5)	80 (6.9)	64 (6.5)	62 (6.4)	14 (6.6)	305 (7.9)
Gender									
Male	845 (53.1)	319 (52.7)	219 (50.7)	224 (54.2)	555 (48.1)	526 (53.4)	515 (53.0)	111 (52.4)	2327 (50.4)
Female	746 (46.9)	286 (47.3)	213 (49.3)	189 (45.8)	600 (52.0)	460 (46.7)	456 (47.0)	101 (47.6)	2290 (49.6)
Birthweight (mean ± SD)	3280 ± 494	3322 ± 487	3321 ± 468	3332 ± 468	3331 ± 501	3254 ± 498	3251 ± 498	3268 ± 521	3185 ± 563
Renal volume (cm ^3^)	-	9.64 ± 2.81	9.62 ± 2.81	9.63 ± 2.84	9.75 ± 2.77	-	-	-	-
Age at renal measurement, years	9.4 ± 1.0	8.8 ± 0.9	8.9 ± 0.9	8.8 ± 0.9	-	9.7 ± 1.0	9.7 ± 1.0	9.7 ± 0.9	-

*Non-response for the BiB renal ultrasound cohort were those who had a foetal renal ultrasound at baseline but did not provide blood or urine sample for follow-up. Non-response for the BiB extended renal follow-up were those who had no renal ultrasound at baseline, participated in the Growing up follow-up but did not provide either blood or urine samples for renal analysis.

In total, 986 provided samples (largely blood) in the BiB extended follow-up group, and for response were compared to those who participated in the follow-up but did not provide blood samples. The response rate in both groups was higher in South Asians and in boys, but there was no difference in birthweight or renal volume between responders and non-responders ( Table 1). 

Of the 1591 participants, 69.1% were South Asian and 40.1% had further and higher maternal education ( Table 2). Mean maternal age was 28.0 years (standard deviation (SD) 5.5) and mean maternal BMI at antenatal booking was 26.3 kg/m ^2^ (SD 5.7). 10% of mothers smoked during pregnancy and 11% reported consuming alcohol. Compared to White European mothers, South Asian mothers were more likely to: be older; be married; to own their own home, be of lower educational attainment and higher parity, and less likely to work, smoke or drink alcohol during pregnancy. A higher percentage had gestational diabetes. Renal volume, birthweight and renal volume adjusting for birthweight were significantly lower in South Asian babies.

**Table 2.** Demographic and clinical details of mothers and children.

Variables	All renal follow-up	Ethnic group		p-value
		White	South Asian	
n	1591	492	1099	
**Mother’s details**				
Mother’s ethnic group				
White	492 (30.9)	492 (100)	-	-
South Asian	1099 (69.1)	-	1099 (100)	-
Educational attainment				
None (<5 GCSE or equivalent)	392 (24.7)	79 (16.1)	313 (28.5)	<0.001
School (≥5 GCSE or equivalent)	454 (28.6)	139 (28.3)	315 (28.7)	
Further (A level) and higher	638 (40.1)	226 (46.0)	412 (37.5)	
Others (other, overseas, unknown)	106 (6.7)	47 (9.6)	59 (5.4)	
% missing	1 (0.1)	1 (0.2)	-	
Marital status				
Married	1203 (80.3)	216 (43.9)	987 (98.0)	<0.001
Single (never married, divorced or separated)	296 (10.8)	276 (56.1)	20 (2.0)	
% missing	92 (5.8)	-	92 (8.4)	
Housing tenure				
Buying/own	1006 (67.1)	304 (61.8)	702 (69.7)	0.002
Renting or other related	493 (32.9)	188 (38.2)	305 (30.3)	
% missing	92 (5.8)	-	92 (8.4)	
Employment status during pregnancy				
Employed	599 (40.0)	350 (71.1)	249 (24.8)	<0.001
Previously employed	386 (25.8)	118 (24.0)	268 (26.6)	
Never employed	513 (34.3)	24 (4.9)	489 (48.6)	
% missing	93 (5.8)	-	93 (8.5)	
Age at antenatal booking, years (mean ± SD)	28.0 ± 5.5	27.6 ± 6.0	28.2 ± 5.2	0.06
% missing	26 (1.6)	7 (1.4)	19 (1.7)	
BMI at antenatal booking (kg/m2) (mean ± SD)	26.3 ± 5.7	27.5 ± 6.3	25.7 ± 5.3	<0.001
% missing	162 (10.2)	25 (5.1)	137 (12.5)	
Parity				
0	521 (33.9)	213 (44.4)	308 (29.1)	<0.001
1	437 (28.4)	177 (36.9)	260 (24.6)	
2	295 (19.2)	62 (12.9)	233 (22.0)	
3+	285 (18.5)	28 (5.8)	257 (24.3)	
% missing	53 (3.3)	12 (2.4)	41 (3.7)	
Mother smoked during pregnancy	153 (10.2)	124 (25.3)	29 (2.9)	<0.001
% missing	93 (5.8)	1 (0.2)	92 (8.4)	
Mother drank alcohol during pregnancy	169 (11.3)	163 (33.1)	6 (0.6)	<0.001
% missing	93 (5.8)	-	93 (8.5)	
Gestational diabetes	140 (8.9)	17 (3.5)	123 (11.3)	<0.001
% missing	19 (1.2)	7 (1.4)	12 (1.1)	
**Child renal measures (scan)**				
Volume (cm ^3^) (mean ± SD deviation)	9.64 ± 2.81	10.63 ± 2.96	8.84 ± 2.40	<0.001
Volume/birth weight (cm ^3^/kg) (mean ± SD)	2.92 ± 0.78	3.07 ± 0.81	2.79 ± 0.73	<0.001
**Child’s details**				
Gender				
Male	845 (53.1)	242 (49.2)	603 (54.9)	0.04
Female	746 (46.8)	250 (50.8)	496 (45.1)	
Gestational age at birth (weeks) (mean ± SD)	39.8 ± 1.2	40.0 ± 1.2	39.7 ± 1.2	<0.001
% missing	16 (1.0)	6 (1.2)	10 (0.9)	
Birth weight (mean ± SD)	3281 ± 494	3473 ± 490	3195 ± 472	<0.001
% missing	16 (1.0)	6 (1.2)	10 (0.9)	
Blood pressure (BP)				
Systolic (sBP)	113 ± 13	113 ± 13	113 ± 13	0.48
Diastolic (dBP)	72 ± 10	70 ± 9	72 ± 10	<0.001
% missing	58 (3.6)	14 (2.8)	44 (4.0)	
Age at renal measurement, years (mean ± SD)	9.4 ± 1.0	9.3 ± 1.1	9.4 ± 1.0	0.08
% missing	4 (0.3)	2 (0.4)	2 (0.2)	
Cystatin C (mg/L)				
n	1257	392	865	
Mean ± SD	0.84 ± 0.09	0.84 ± 0.08	0.84 ± 0.09	0.42
eGFR (Schwartz creatinine only) *				
n	1291	369	922	0.002
Mean ± SD	116 ± 17	113 ± 16	116 ± 18	
<60	1 (0.1)	1 (0.2)	-	
eGFR (Schwartz combined formula) **				
N	1156	353	803	
Mean ± SD	92 ± 9	91 ± 9	92 ± 9	0.007
<60	1 (0.1)	-	1 (0.1)	
eGFR (Zappitelli CystatinC) ***				
N	1257	392	865	
Mean ± SD	94 ± 11	94 ± 10	94 ± 11	0.52
<60	1 (0.1)	-	1 (0.1)	
eGFR (Filler CystatinC) ****				
n	1257	392	865	
Mean ± SD	149 ± 37	150 ± 36	149 ± 38	0.59
<60	4 (0.3)	-	4 (0.4)	
Albumin creatinine ratio (ACR)				
n	595	223	372	
Mean ± SD	0.7 ± 2.2	0.6 ± 1.0	0.7 ± 2.6	0.81
≥2.5 (M)/ ≥3.5 (F)	14 (2.4)	7 (3.1)	7 (1.9)	
Protein creatinine ratio (PCR)				
n	553	207	346	
Mean ± SD	10.1 ± 4.3	9.9 ± 2.7	10.2 ± 5.0	0.53
≥15	30 (5.4)	10 (4.8)	20 (5.8)	
Urinary retinol binding protein (RBP) (mg/L)				
<2	620 (99.2)	231 (99.6)	389 (99.0)	0.59
3	2 (0.3)	-	2 (0.5)	
Missing/No result	3 (0.5)	1 (0.4)	2 (0.5)	

SD = Standard deviation, eGFR = Estimated Glomerular Filtration Rate.

*Schwartz creatinine only eGFR = 41.3*(height (m)/ S _cr_)

**Schwartz combined eGFR = 39.8 x [(height (m)/S _cr_(mg/dl)] ^0.456 ^x [1.8/cystatin C (mg/L)] ^0.418 ^x [30/blood urea nitrogen (mg/dl)] ^0.079^ x [1.076 ^male^] x [height (m)/1.4] ^0.179^

***Zappitelli eGFR = 75.94/[cystatinc^1.17]

****Filler log(eGFR) = 1.962 + (1.123*log(1/cystatinc))

Mean eGFR was 92 ml/min/1.73m ^2^ (SD 9) using Schwartz combined and 94 ml/min/1.73m ^2^ (SD 11) using Zappitelli ( Table 2). CKD prevalence was rare (1 with eGFR <60 ml/min/1.73m ^2^, 14 (2.4%) with ACR ≥2.5 mg/mmol in boys/3.5 mg/mmol in girls). RBP results were within the normal range for the whole sample. Mean eGFR was slightly higher in South Asian children when using creatinine formulae, but no difference was seen in Cystatin C or Cystatin C based eGFR. Mean ACR and PCR were similar in both ethnic groups. sBP was similar in both ethnic groups but mean dBP was slightly higher in South Asians (72 mmHg, SD 10) than in White Europeans (70 mmHg, SD 9) and the difference was significant (p<0.001). sBP had a small negative correlation with cystatin C eGFR (-0.12) but all other correlation estimates of BP and eGFR/ACR/PCR were <0.1.

Whilst South Asian children had higher eGFR than White European children using creatinine-based formulae and a higher dBP, these findings were only significant in the unadjusted models and the socio-demographic model for dBP ( Table 3). There were no other significant associations between ethnicity and Cystatin C eGFR, ACR, PCR and sBP at 7–11 years in any model. eGFR estimates were similar when child BMI was adjusted for instead of BSA ( *Extended data,* Supplementary Table 1 ^39^).

**Table 3.** Summary table of differences in kidney function, kidney damage and blood pressure by ethnicity: univariable and multivariable.

Model variable	Schwartz eGFR creatinine only equation *	Schwartz eGFR combined equation *	Zappitelli eGFR cystatin C only equation *	Filler eGFR cystatin C only equation *	Cystatin C	Albumin creatinine ratio (ACR)		Protein creatinine ratio (PCR) (logged)	Systolic blood pressure (sBP)	Diastolic blood pressure (dBP)
						Detectable	Result			
	Beta estimate (95% CI)	Beta estimate (95% CI)	Beta estimate (95% CI)	Beta estimate (95% CI)	Beta estimate (95% CI)	Odds ratio (95% CI)	Beta estimate (95% CI)	Beta estimate (95% CI)	Beta estimate (95% CI)	Beta estimate (95% CI)
**Unadjusted – n**	1291	1156	1257	1257	1257	595	296	553	1533	1533
White ethnicity	Ref	Ref	Ref	Ref	Ref	Ref	Ref	Ref	Ref	Ref
South Asian ethnicity	**3.36** **1.27 to 5.44**	**1.55** **0.42 to 2.67**	-0.42 -1.71 to 0.86	-1.24 -5.70 to 3.22	0.004 -0.006 to 0.015	0.84 0.60 to 1.17	0.15 -0.25 to 0.56	0.005 -0.04 to 0.05	0.50 -0.89 to 1.89	**2.04** **0.99 to 3.10**
**Socio-demographic - n**	1202	1079	1177	1177	1177	568	284	530	1443	1443
South Asian ethnicity	2.49 -0.70 to 5.67	1.08 -0.65 to 2.80	-1.01 -2.91 to 0.89	-3.59 -10.16 to 2.98	0.008 -0.008 to 0.023	0.92 0.56 to 1.51	-0.01 -0.32 to 0.29	-0.03 -0.10 to 0.03	-1.60 -3.60 to 0.40	**2.09** **0.55 to 3.63**
**Maternal/pregnancy – n**	1112	998	1090	1090	1090	531	268	495	1340	1340
South Asian ethnicity	2.60 -1.00 to 6.19	1.32 -0.60 to 3.25	-1.10 -3.23 to 1.03	-4.00 -11.37 to 3.38	0.008 -0.010 to 0.025	0.69 0.40 to 1.22	0.11 -0.22 to 0.44	-0.02 -0.10 to 0.05	-1.76 -3.98 to 0.47	1.54 -0.17 to 3.25
**Fully adjusted model - n**	1071	967	1014	1014	1014	489	246	455	1265	1265
South Asian ethnicity	2.58 -1.08 to 6.24	1.32 -0.65 to 3.30	-0.25 -2.48 to 1.98	-1.18 -8.89 to 6.53	0.001 -0.018 to 0.019	0.72 0.39 to 1.32	0.04 -0.28 to 0.36	-0.03 -0.11 to 0.05	-2.18 -4.47 to 0.11	0.88 -0.90 to 2.65

*estimated Glomerular Filtration Rate (eGFR) Socio-demographic model is adjusted for maternal age, maternal educational attainment, housing tenure, employment status and marital status, all at pregnancy and child age at kidney function measurement Maternal/pregnancy model is socio-demographic model plus maternal BMI, parity, alcohol consumption in pregnancy, maternal smoking in pregnancy, and gestational diabetes Fully adjusted model is maternal/pregnancy model plus birthweight, child body surface area (for kidney outcomes) or body mass index (for blood pressure) and child gender (all outcomes), and systolic and diastolic blood pressure (for eGFR, cystatin C, ACR and PCR only). Complete results of the Fully adjusted models is shown in *Extended data,* Supplementary Table 2 ^39^.

Adjusting for renal volume in the BiB renal ultrasound follow-up subsample did not affect the estimates ( Table 4). There was little difference when measured height was used regardless of timing between blood sample and height measurement or when the sample was restricted to the subset with blood sample and height measurement at the same time ( *Extended data,* Supplementary Table 3 ^39^).

**Table 4.** Summary table of differences in kidney function, kidney damage and blood pressure by ethnicity in the subset with renal ultrasound scans at 34 weeks gestation: univariable and multivariable adjusting for renal volume.

Model variable	Schwartz eGFR creatinine only equation *	Schwartz eGFR combined equation *	Zappitelli eGFR cystatin C only equation *	Filler eGFR cystatin C only equation *	Cystatin C	Albumin creatinine ratio (ACR) *		Protein creatinine ratio (PCR) (logged)	Systolic blood pressure (sBP)	Diastolic blood pressure (dBP)
						Detectable	Result			
	Beta estimate (95% CI)	Beta estimate (95% CI)	Beta estimate (95% CI)	Beta estimate (95% CI)	Beta estimate (95% CI)	Odds ratio (95% CI)	Beta estimate (95% CI)	Beta estimate (95% CI)	Beta estimate (95% CI)	Beta estimate (95% CI)
**Unadjusted – n**	364	345	409	409	409	399	204	375	577	577
White ethnicity	Ref	Ref	Ref	Ref	Ref	Ref	Ref	Ref	Ref	Ref
South Asian ethnicity	2.99 -0.57 to 6.55	0.88 -1.00 to 2.76	-1.04 -3.02 to 0.95	-3.47 -10.45 to 3.52	0.008 -0.007 to 0.023	0.90 0.61 to 1.34	0.28 -0.27 to 0.84	0.04 -0.01 to 0.10	0.75 -1.24 to 2.73	1.34 -0.24 to 2.92
**Fully adjusted ** **model - n**	333	317	364	364	364	339	173	316	520	520
South Asian ethnicity	2.90 -3.01 to 8.81	1.03 -2.05 to 4.11	-0.08 -3.30 to 3.16	-0.25 -11.66 to 11.15	-0.0001 -0.025 to 0.025	0.96 0.46 to 2.02	0.04 -0.27 to 0.35	-0.01 -0.11 to 0.10	0.02 -3.07 to 3.10	2.41 -0.01 to 4.89

*eGFR = estimated Glomerular Filtration Rate Fully adjusted model is adjusted for maternal age, maternal educational attainment, housing tenure, employment status and marital status, all at pregnancy; maternal BMI, parity, alcohol consumption in pregnancy, maternal smoking in pregnancy, gestational diabetes, birthweight, child body surface area (for kidney outcomes) or body mass index (for blood pressure), child gender, and renal volume (measured using ultrasound scans at 34 weeks of gestation) (all outcomes) and additionally for systolic and diastolic blood pressure (for eGFR, cystatin C, ACR and PCR only).

## Discussion

South Asian adults are at high risk of premature severe CKD. Prevention of adult CKD may be informed by better understanding of the early origins and lifecourse determinants. Using BiB data, we have previously shown that South Asian infants have smaller kidneys compared with white European infants ^25^.

In this study whilst unadjusted models found higher dBP and higher creatinine-based eGFR in South Asian children, there were no significant ethnic differences in adjusted models on renal function or BP at 7–11 years. These results suggest that any impact of reduced foetal renal volume was not biologically significant on kidney function by this age or alternatively that there was compensation for the presumed reduced nephron number at birth by hyperfiltration ^19^. It is therefore possible that changes may manifest later in development, hence the need for further follow-up.

Published data on ethnic differences in childhood kidney function are very limited. Ethnic differences in kidney volume and function in various ethnic groups (which did not include those from the Indian subcontinent) were found in the Generation R cohort in the Netherlands. Compared to Dutch children, Moroccan and Turkish children aged 6 had a higher eGFR whereas Dutch Antillean and Surinamese-Creole children had a lower eGFR ^40^. Surinamese Hindustani children had lower kidney volume. The authors concluded that whilst these differences were not clinically significant (i.e. requiring nephrology referral), they may track and alter adult CKD risk.

The BP findings extends BiB findings of higher dBP in South Asian Pakistani children aged 4–5 years, which was not related to socioeconomic factors ^27, 28^. Ethnic differences in BP were found in the Generation R cohort with Cape Verdean and Turkish children having higher sBP and dBP compared to Dutch children ^40^. In a review, higher dBP in Pakistani children was found in the 1999 Health Survey for England (5–15 years) though not in three other studies where ethnicity was combined as South Asian; but was also found in a cross-sectional study in England at 9–10 years and in a longitudinal school-based cohort in London at 14–16 years (though not at 11–13 years) ^41– 43^.

Our study strengths include a large predominantly bi-ethnic population from the same city, availability of baseline foetal and maternal data on covariates, a subsample with detailed ultrasonography with high reliability in pregnancy for renal measures and first morning urine sample taken to measure albuminuria and proteinuria. We measured both creatinine and cystatin C so could compare the findings using the different formulae, all have been validated in paediatric populations. eGFR calculated using the Schwartz creatinine only formula was higher than that using the Zappitelli cystatin C, similar to other studies ^44, 45^. eGFR calculated using the Filler formula was the highest which has been observed previously ^44^. Cystatin C formulae may be more sensitive than creatinine formulae for evaluating kidney function which has been suggested previously given it is freely filtered by glomeruli, fully catabolised by renal tubules and not excreted by non-renal routes ^46^. As in adults there is no correction for ethnicity in eGFR formulae.

There were limitations to the study. Uptake to follow-up was lower than expected in the renal ultrasound sub-study, and we had to supplement with recruitment opportunistically from those providing a blood sample. This limited our power to study renal volume effects and led to the inclusion of a sample with higher levels of missing baseline data as a proportion of mothers had not filled out the baseline questionnaire. We did not attempt to impute missing values as we had limited data on such individuals. The lower than expected uptake highlights the challenge of undertaking follow-up in a population with ethnic diversity with high levels of socio-economic disadvantage, especially with invasive testing. There was a difference in timing between anthropometric measurements and blood sampling in some children which might have affected the creatinine based eGFR measurement though we used growth centile charts to take this into account, and sensitivity analysis showed no difference. However, we had a slightly higher proportion of South Asian children (54%) than White children (40%) with a difference in timing between the blood sample and height measurement. The growth centile charts are for the UK general population and may overestimate eGFR in South Asian children. Our assessment of kidney function and BP were based on a single measure as in other studies and any variation is likely to reduce the strength of any associations. We had no valid measures of kidney volume in childhood as this was not practically feasible.

We found lower levels of microalbuminuria (2.5%) than in the Generation R cohort (7.1%) in children followed up at median age of 5.9 years (95% range, 5.7 to 6.6) using the same cut-offs to define microalbuminuria ^47^. The prevalence of albuminuria in children aged 5–18 years from the Australian Health Survey 2011–2013 was 12.8% and higher in girls (15.5%) than in boys (10.2%) ^48^. A population-based cross-sectional study in Australia (2015–16) reported a higher prevalence of albuminuria (15.1% overall, 20.8% in girls and 10.1% in boys) in children aged 11–12 years ^49^. The cut-off used to define albuminuria in both Australian studies was different (ACR >3.4 mg/mmol ACR). All three studies used a random urine sample whereas we used the first morning urine sample. The lower prevalence in our study is unexplained.

In conclusion, there was no evidence of significant ethnic differences in kidney function at pre-pubertal age despite the lower kidney volume at birth in South Asian babies in those with foetal renal ultrasound scans. However South Asian children may be at higher long-term CKD risk through reduced foetal kidney volume with compensatory hyperfiltration, higher BP, and greater propensity to adverse metabolic patterns. Promoting kidney development through childhood and adolescence, for example by aiming for normal weight trajectory and obesity avoidance would be relevant for all children but especially those with higher adult CKD risk such as South Asians. Further longitudinal follow-up is required in the BiB cohort through adolescence when any propensity to metabolic risk becomes more apparent, measuring kidney function and ideally size, to see how kidney function and BP measures track, and to determine if there are emerging differences by ethnicity. This would inform population strategies and risk stratification to prevent adult CKD.

## Data Availability

Scientists are encouraged to make use of the BiB data, which are available through a system of managed open access. Before you contact BiB, please make sure you have read our Guidance for Collaborators. Our BiB executive review proposals on a monthly basis and we will endeavour to respond to your request as soon as possible. If you are unsure if we have the data that you need please contact a member of the BiB team ( borninbradford@bthft.nhs.uk). Once you have formulated your request please complete the ‘Expression of Interest’ form and email the BiB research team ( borninbradford@bthft.nhs.uk). If your request is approved, we will ask you to sign a collaboration agreement; if your request involves biological samples, we will ask you to complete a material transfer agreement. Open Science Framework: Supplementary tables for: Ethnic differences in kidney function in childhood: the Born in Bradford Cohort Renal Study. https://doi.org/10.17605/OSF.IO/MZHAK ^39^. Data are available under the terms of the Creative Commons Zero "No rights reserved" data waiver (CC0 1.0 Public domain dedication).
