# Supplementary material for: Ethnic differences in kidney function in childhood: the Born in Bradford Cohort Renal Study
Source: Wellcome Open Res. 2023 Aug 25;7:112. Originally published 2022 Mar 25. [Version 2] doi: 10.12688/wellcomeopenres.17796.2 (PMC10233317; doi:10.12688/wellcomeopenres.17796.2)
Supplement: Peer review history

# Reviewer report: Reviewer response for version 2

**DOI:** 10.21956/wellcomeopenres.22083.r65845

**Published:** 2023-10-18

**Authors:** Midori Awazu

**Affiliations:** 1 Tokyo Metropolitan Ohtsuka Hospital, Tokyo, Japan

The manuscript is basically unchanged. My previous comments regarding the calculation of eGFR in South Asian children and the use of BP index have not been addressed. The authors have not described the limitations of the study on these points either.

 For example I previously pointed out the presence of the paper on eGFR formulae in South Asian adults, yet the authors have kept the following description;

 “As in adults there is no correction for ethnicity in eGFR formulae.”

 They have described the method of BP measurement, but what is the rationale for the following?

 “One BP measurement was recorded per child consistent with other studies undertaken within a school setting.”

Is the work clearly and accurately presented and does it cite the current literature?

No

If applicable, is the statistical analysis and its interpretation appropriate?

Partly

Are all the source data underlying the results available to ensure full reproducibility?

Yes

Is the study design appropriate and is the work technically sound?

No

Are the conclusions drawn adequately supported by the results?

Partly

Are sufficient details of methods and analysis provided to allow replication by others?

No

Reviewer Expertise:

Developmental origins of health and disease, pediatric nephrology, development, blood pressure

I confirm that I have read this submission and believe that I have an appropriate level of expertise to confirm that it is of an acceptable scientific standard, however I have significant reservations, as outlined above.

# Reviewer report: Reviewer response for version 2

**DOI:** 10.21956/wellcomeopenres.22083.r65846

**Published:** 2023-08-31

**Authors:** Jerome Harambat

**Affiliations:** 1 Bordeaux University Hospital, Bordeaux, France

The authors responded well to the comments of the reviewers, I have no further comments.

Is the work clearly and accurately presented and does it cite the current literature?

Yes

If applicable, is the statistical analysis and its interpretation appropriate?

Yes

Are all the source data underlying the results available to ensure full reproducibility?

Yes

Is the study design appropriate and is the work technically sound?

Yes

Are the conclusions drawn adequately supported by the results?

Yes

Are sufficient details of methods and analysis provided to allow replication by others?

Yes

Reviewer Expertise:

Pediatrics, Kidney disease, epidemiology

I confirm that I have read this submission and believe that I have an appropriate level of expertise to confirm that it is of an acceptable scientific standard.

# Reviewer report: Reviewer response for version 1

**DOI:** 10.21956/wellcomeopenres.19697.r56930

**Published:** 2023-05-31

**Authors:** Daniel Landau

**Affiliations:** 1 Tel Aviv University, Tel Aviv, Israel

The authors report on a longitudinal data of mixed ethnicity (South Asians Vs White British) birth cohort in England, concentrating on kidney volume  at 34 weeks gestation (absolute and corrected for birth weight)  and kidney function as assessed during school age, including serum creatinine, cystatin C and urine albumin. The authors report that even though birth weight corrected kidney volume was lower in children of South Asian ancestry. kidney function was not significantly differentץ The overall rate of detection of permanent kidney damage in this study was low  ( only 1 with eGFR <60 ml/min/1.73m2, 14 (2.4%) with ACR ≥2.5 mg/mmol in boys/3.5 mg/mmol in girls).

 The study reflects a huge effort, and its results are worth a indexing. My concern remains on the methodology, regarding mostly: 

1. Selection bias: only < 2000 out of 13000 pregnancies have been originally followed since pregnancy. 
2. The accuracy of a fetal US (which may be unable to pick the kidneys at their maximal longitudinal diameter) to assess true kidney volume. 

Specific critiques:  

 Page 5 :"Child height required to calculate eGFR... was measured at the same visit as the blood sample in 52.0%, the height measurement was standardised to time of sample using centile charts if not measured at the same visit."  Please comment on the possible bias of this analytical decision, specially. related to: 

1. Are there different normal growth charts for White British and South Asians?
2. Could there be more South Asians than White British with missing heights?
3. Did the authors consider using the new height independent child eGFR equations developed by Pottel et al. ? 

Minor critiques: 

 Page 3 : "Women who were attending for the OGTT at 26–28 weeks of gestation... etc". Please clarify this abbreviation when first used: is it "oral glucose tolerance test"?

Is the work clearly and accurately presented and does it cite the current literature?

Yes

If applicable, is the statistical analysis and its interpretation appropriate?

I cannot comment. A qualified statistician is required.

Are all the source data underlying the results available to ensure full reproducibility?

Yes

Is the study design appropriate and is the work technically sound?

Yes

Are the conclusions drawn adequately supported by the results?

Partly

Are sufficient details of methods and analysis provided to allow replication by others?

Yes

Reviewer Expertise:

Clinical pediatric nephrology, epidemiology of childhood CKD, genetic renal diseases

I confirm that I have read this submission and believe that I have an appropriate level of expertise to confirm that it is of an acceptable scientific standard, however I have significant reservations, as outlined above.

# Response

**Published:** 2023-08-18

**Authors:** Nida Ziauddeen

**Affiliations:** University of Southampton, UK

1. >6000 of the original cohort were followed up at 7-11 years. The renal study was undertaken in a sub-sample and the sample size of 1591 are the participants that provided a blood or urine sample for renal analysis. We assessed difference in sample characteristics at follow-up using selected descriptive variables at baseline (presented in Table 1). There was no obvious major response bias but limited our statistical power as uptake to the follow-up component was lower than expected. The low uptake highlights the challenge of undertaking follow-up in a population with high levels of socio-economic disadvantage and ethnic diversity, especially with invasive testing. We have stated this as a limitation.
2. The fetal ultrasounds were performed by experienced sonographers and intra-observer reliability was high. We are unaware what study the reviewer is referring to regarding the accuracy of the fetal ultrasound and would appreciate if the reviewer could direct us to this.

Specific critiques:  

1. No, the growth chart used is for the UK general population and used in clinical practice. However, this could overestimate eGFR and we have added this as a limitation.
2. All children had a recorded height measurement. There was a slightly higher proportion of South Asian children (54%) than White children (40%) with a difference in timing between the blood sample and height measurement which may have introduced bias due to the greater use of growth charts in south Asian.
3. Thank you for the suggestion of height independent eGFR equations. This equation requires a population median serum creatinine which we do not have. We have taken a similar approach and used equations used in other literature that we have cited.

Minor critiques: 

 Thank you, we have clarified the abbreviation at first use in the first paragraph under the methods section.

# Reviewer report: Reviewer response for version 1

**DOI:** 10.21956/wellcomeopenres.19697.r56927

**Published:** 2023-05-16

**Authors:** Midori Awazu

**Affiliations:** 1 Tokyo Metropolitan Ohtsuka Hospital, Tokyo, Japan

The study aimed to investigate ethnic differences in kidney function and blood pressure (BP) in early childhood, specifically comparing South Asian and White European children. In 1591 children aged 7-11 years, estimated glomerular filtration rate (eGFR), urine albumin to creatinine ratio, and BP were assessed. The results showed no significant ethnic differences in kidney function at pre-pubertal age, despite previous findings of smaller fetal kidney size in South Asian children. Diastolic BP was slightly higher in South Asian children.

 The aim of the study is important and the methods are appropriate except for the following two points.

1. In adult South Asians, eGFRCr was reported to overestimate measured GFR, and the modified equation has been created (Jessani, Am J Kidney Dis, 2014) ^1^. eGFRcys, on the other hand, underestimates measured GFR (Wang Y, Kidney Int Rep, 2021). In view of these facts, the use of Schwarz or Zappitelli formulae in South Asian children cannot be justified. The modified formulae to estimate eGFR in South Asian children is needed.
  Page 13, right first line;
  “As in adults there is no correction for ethnicity in eGFR formulae.“
  This statement is erroneous. It is currently not available in children but is needed.
  The higher creatinine-based eGFR in South Asian children than White European children is in fact contrary to what is expected. The authors speculate this to be due to compensation. Overshooting by compensation (higher eGFR than White European children), however, is unlikely.
2. Regarding BP measurement, the authors do not state which measurement value (before or after the skinfold measurement or the average of the two) was used. The arm width is expected to be variable with the age of the study subjects (7-11 years). Was the right cuff size selected? Also, as the authors stated, the BP was measured on one occasion only. The difference of diastolic pressure between South Asian and White European children is subtle and could result from small variations during BP measurements.
3. One more important point is that in children BP is assessed according to reference values by gender, age, and height. Therefore BP index (BP divided by 95th percentile BP for each patient) not raw values should be used for comparison.

Is the work clearly and accurately presented and does it cite the current literature?

No

If applicable, is the statistical analysis and its interpretation appropriate?

Partly

Are all the source data underlying the results available to ensure full reproducibility?

Yes

Is the study design appropriate and is the work technically sound?

No

Are the conclusions drawn adequately supported by the results?

Partly

Are sufficient details of methods and analysis provided to allow replication by others?

No

Reviewer Expertise:

Developmental origins of health and disease, pediatric nephrology, development, blood pressure

I confirm that I have read this submission and believe that I have an appropriate level of expertise to confirm that it is of an acceptable scientific standard, however I have significant reservations, as outlined above.

## References

[1] : Estimation of GFR in South Asians: a study from the general population in Pakistan. *Am J Kidney Dis* .2014;63(1) : 10.1053/j.ajkd.2013.07.023 49-58 10.1053/j.ajkd.2013.07.023 24074822PMC4428680

[2] : Performance and Determinants of Serum Creatinine and Cystatin C-Based GFR Estimating Equations in South Asians. *Kidney Int Rep* .2021;6(4) : 10.1016/j.ekir.2021.01.005 962-975 10.1016/j.ekir.2021.01.005 33912746PMC8071622

# Response

**Published:** 2023-08-18

**Authors:** Nida Ziauddeen

**Affiliations:** University of Southampton, UK

1. Thank you for the suggested references but both are in adults. No formulae are available for children that takes account of ethnicity. While we agree that the different formulae may under- or over-estimate eGFR and that a correction may need to be applied in South Asians, no reliable correction is available for children. This paper ( https://pubmed.ncbi.nlm.nih.gov/29492066) compared updated Schwartz, original Schwartz and height independent formula to look at eGFR in Pakistani children. Although the sample was malnourished children, the authors found no differences between the different formulae. We recognize there are challenges with different methods of measuring eGFR but we based our approach on those that are well recognised in the literature.  

 2. Thank you, the average of the two measurements were taken and the appropriate cuff size was selected. We have added this to the methods section.

 3. Thank you, we are not categorising the individuals as hypertensive where you do need reference values. We followed others ( https://pubmed.ncbi.nlm.nih.gov/29895845/ https://www.ncbi.nlm.nih.gov/pmc/articles/PMC7612360/),  who have used actual blood pressure values.

# Reviewer report: Reviewer response for version 1

**DOI:** 10.21956/wellcomeopenres.19697.r52088

**Published:** 2022-09-30

**Authors:** Jerome Harambat

**Affiliations:** 1 Bordeaux University Hospital, Bordeaux, France

This report is a birth cohort study from the UK (BiB) assessing ethnic differences in kidney outcomes during childhood.

 The authors investigated whether South Asian children in the UK who are known to have lower birthweight and kidney volume a birth than White Europeans are at higher risk of early kidney disease at age 7-11.

 Out of >13,000 children enrolled in the BiB birth cohort, about 1,600 were included in this analysis, of whom a subgroup of 600 had a renal ultrasound follow-up at 8-11 years. In multivariable analyses, there was no differences in various kidney outcomes (eGFR, BP, albuminuria) between the South Asian and White European populations. In the subsample with renal ultrasound follow-up, the significant ethnic difference seen in kidney volume at pre-pubertal age did no translate into differences in kidney function or blood pressure. The authors concluded that despite no apparent kidney impairment at 7-11 years, South Asians children may be at higher long-term CKD risk due to reduced nephron mass so longitudinal follow-up remains necessary.

 The paper is very well written, the BiB birth cohort well conducted and the methodology is sound. The flow chart and the DAG are appropriate and the conclusion is supported by the data shown.

 I have a few minor comments:

1. The study population include 1591 children that is 11% of the BiB birth cohort. How did the authors assess for and handle potential sample selection bias?
2. The authors sometimes used the term mediation. For instance, the objective in the introduction is whether differences in kidney function and BP in childhood are mediated by fœtal kidney size. A mediation analysis method would have been appropriate in this study, but a tradtional multivariable linear regression was performed, which is fine. I would recommend to avoid the term mediation or to consider a true mediation analysis.
3. The directed acyclic graph is rather convincing and well done but remains complex, and the rationale of adding covariates sequentially in 3 distinct models is not completely clear to me. Perhaps a more parsimonious and straightforward model focusing on the main determinants would fulfil the message just as well.
4. The authors mentionned a mean eGFR using Schwartz combined of 92 ml/min/1.73 m² and only 1 child with an eGFR < 60. How many patients would have an eGFR of 60-89 ml/min/1.73 m², and how many have a BP > 95th centile?
5. Could you show in supplementary data the kidney function and BP in the subgroup of patients with actual small volume kidney (for example kidney volume or length < - 2 SDS) irrespective of ethnicity?
6. Could the authors comment on the public health message to take up in this study in addition to tracking kidney function and BP in the BiB cohort?

Is the work clearly and accurately presented and does it cite the current literature?

Yes

If applicable, is the statistical analysis and its interpretation appropriate?

Yes

Are all the source data underlying the results available to ensure full reproducibility?

Yes

Is the study design appropriate and is the work technically sound?

Yes

Are the conclusions drawn adequately supported by the results?

Yes

Are sufficient details of methods and analysis provided to allow replication by others?

Yes

Reviewer Expertise:

Pediatrics, Kidney disease, epidemiology

I confirm that I have read this submission and believe that I have an appropriate level of expertise to confirm that it is of an acceptable scientific standard.

# Response

**Published:** 2023-08-18

**Authors:** Nida Ziauddeen

**Affiliations:** University of Southampton, UK

1. We assessed difference in sample characteristics at follow-up using selected descriptive variables at baseline (presented in Table 1). There was no obvious major response bias but limited our statistical power as uptake to the follow-up component was lower than expected. The low uptake highlights the challenge of undertaking follow-up in a population with high levels of socio-economic disadvantage and ethnic diversity, especially with invasive testing. We have stated this as a limitation.
2. Thank you, we have removed the term mediation from the paper.
3. Thank you. We added covariates sequentially in terms of the types of determinants (socio-demographic, maternal/pregnancy factors and child factors) with model 3 being the fully adjusted model including all the covariates from the DAG.
4. 476 children with eGFR of 60-89 ml/min/1.73. We haven’t presented the BP >95th centile as not sure what the benefit would be.
5. Thank you for this suggestion. We have looked into this and unfortunately the numbers are too small for us to be able to present this.
6. Our findings did not identify any ethnic specific action per se but we have added the following sentence to the conclusion paragraph: promoting kidney development through childhood and adolescence, for example by aiming for normal weight trajectory and obesity avoidance  would be relevant for all children but especially those with higher adult CKD risk such as south Asians.
